# Progress in the clinical treatment of keloids

**DOI:** 10.3389/fmed.2023.1284109

**Published:** 2023-11-16

**Authors:** Wenli Qi, Xinyue Xiao, Jing Tong, Nengqiang Guo

**Affiliations:** Department of Plastic Surgery, Union Hospital, Tongji Medical College, Huazhong University of Science and Technology, Wuhan, China

**Keywords:** keloids, surgical therapy, physical therapy, drug therapy, biological therapy

## Abstract

Keloid is a pathological scar that is higher than the skin surface following skin damage. Its lesion range often extends beyond the original damage boundary and does not naturally subside over time. Its pathogenesis is very complex, currently the main causes include fibroblast excessive proliferation, collagen and extracellular matrix (Extracellular matrix, ECM) excessive deposition, excessive angiogenesis, and so on. The traditional treatment method primarily involves surgical intervention, but it is associated with a high recurrence rate post-surgery. Consequently, many treatment methods are derived according to the different clinical characteristics of keloid. This paper will review the therapeutic progress in recent years from surgical treatment, physiotherapy, drug therapy, and biological therapy, with the goal of offering valuable insights for the clinical treatment of keloids.

## 1. Introduction

**Figure 1:**
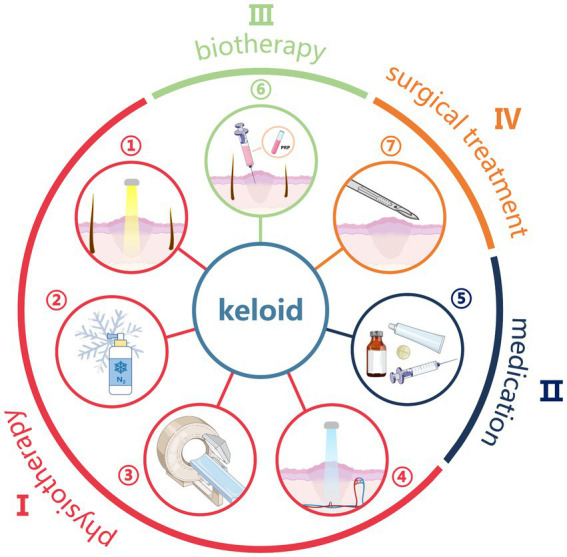
Schematic diagram of keloid treatment methods.

Figure 1 shows the main treatment methods for keloid nowadays, which can be categorized into: physical therapy, drug therapy, biological therapy and surgical therapy, corresponding to serial numbers I, II, III, and IV in the figure, respectively. Physical therapy mainly includes phototherapy (①), cryotherapy (②), radiation therapy (③) and photodynamic therapy (④), which mainly inhibit or improve the formation of scar through the influence of physical factors on fibroblasts, blood vessels and collagen; drugs used in drug therapy (⑤) include hormones, antitumor, botulinum toxin type A, vitamins, and natural plant organics and so on, which mainly regulate the cellular function of the skin by local injection or topical application of drugs; biologic therapies (⑥), including RNA-based therapies, cytokine therapies, enzyme inhibitor therapies, constitutive fat grafting, and platelet-rich plasma therapies, etc., which are mainly used to regulate the repair process of the skin by utilizing biomolecules or cells to inhibit or improve the fibrosis; while surgical treatment (⑦) is suitable for medium to large and mature keloids, and the therapeutic effect is achieved by removing the scar tissue. Of course, according to different clinical characteristics, the above different treatment methods can be reasonably combined to achieve the best therapeutic effect.

Keloid is a pathological scar with an appearance higher than the skin surface and a tough texture, which occurs on the skin surface with high tension, such as facial jaw, ear, chest and back (1). The extent of the lesion will continuously expand into the surrounding normal skin tissue and go beyond the boundary of the original injury, and it will not subside spontaneously over time. There are many factors affecting keloids, most of which come from external factors, such as surgery, trauma, skin infections, burns, acne, herpes zoster, mosquito bites, etc. Additionally, some internal factors caused by personal differences, such as gender, age, bad living habits, formation site, hypertension and other basic diseases and genetic factors will lead to the production of keloids (2). Due to the serious impact on beauty, and may appear itching and exercise limitations and other symptoms, the physical and mental health of patients are greatly affected.

The pathogenesis of keloid is after skin damage will repair any link in the process of abnormal, such as excessive angiogenesis, inflammatory reaction, aggravation of fibroblasts excessive proliferation, can cause ECM excessive production, collagen proportion disorder and collagen disorder, eventually manifested as excessive fibrosis form keloid (3).

In recent years, the keloid treatment method has emerged in an endless stream. However, due to the keloid treatment method with tumor-like characteristics, high recurrence rate and strong personal differences, finding a set of effective treatment plans has also become an important task for clinicians and researchers (4). This paper reviews the clinical treatment status and progress of keloid to provide a reference for clinicians (Figure 1).

## 2. Surgical treatment

Surgical therapy is usually suitable for medium and large keloids and already mature keloids, but postoperative recurrence rates are extremely high, reaching 45–100% (5). It is generally used in combination with postoperative adjuvant therapy, thus reducing the recurrence rate (6).

Excessive skin tension is the main cause of postoperative recurrence (7). After keloid removal, the tension-reduction suture is generally used to prevent recurrence; if the keloid area is too large to be directly stitched, it will be solved by skin dilator and adjacent flap transplantation. In recent years, the more common sutures include: distal buried dermal suture (8), Buried vertical mattress type suture (9), Zhang sutures (10) wait a minute. Under the action of reducing tension and suture, the production of keloid can be greatly reduced, and other auxiliary treatment after surgery can effectively inhibit the recurrence of keloid. Surgical treatment has also become the cornerstone of keloid treatment.

The treatment of keloids is closely related to their size and location and can be specifically divided into preoperative, intraoperative, and postoperative.

### 2.1. Preoperative

The preoperative surgical approach is established and an adequate surgical plan may effectively prevent keloid development. The outer layer is a dense layer of collagen, the middle is a rich vascular network, and the inner layer is a core made up of fibroblasts, with a clear demarcation layer between the superficial skin layer and the papillary layer of the dermi (11). If the keloid is large because direct excision and suturing will lead to an excessive amount of tension on the surface skin, the superficial skin, as well as the subcutaneous vascular network of the keloid, is usually preserved [The skin of the keloid and the subcutaneous vascular network (i.e., the keloid flap)] are typically preserved, with only the nuclear portion of the inner keloid layer excised, which is more common clinically: earlobe keloid (12) and chest keloid (13). Particularly noteworthy is the blood supply to the flap which separates the few penetrating vessels beneath the flap during the process of dissection; direct sectioning of the entire layer will result in the flap becoming a fully layered composite tissue with no vascular supply. The wound will not heal, or the possibility of infection will increase. Proper blood supply allows for better survival of the flap on the one hand and less scar growth on the other (14).

### 2.2. Intraoperatively

Watson et al. suggested that the most important aspect of surgical treatment of keloid is a reasonable incision design and good suturing technique (15).

The design of the surgical incision is a critical factor that significantly influences the wound tension during the proliferative and remodeling phases of scarring, and is closely related to scar prevention. The incision must follow the Relaxed skin tension (RSTL) line. Despite the Lange line and RSTL running in the same direction in several regions of the body, these lines differ significantly in mechanically complex regions (e.g., the corners of the mouth, the external canthus, and the temples), and if there are no such lines in regions of high surface tension, such lines are present, the patient must be told in advance.

Traditional interrupted sutures do not provide prevention of keloids and there is a risk of scar enlargement and spread due to increased wound strain due to motion, body position, or tissue loss (after excision of the lesion). Different sutures can be used intraoperatively to prevent keloids by reducing tension, such as intradermal sutures, hypotonic sutures, super hypotonic sutures, etc. Subcutaneous closure with polypropylene sutures, in conjunction with appropriate compression therapy treatment of the tissue, is the currently more clinically utilized technique. A permanent transparent nylon suture placed deep into the dermis is also effective.

### 2.3. Postoperatively

Silicone scar patches or silicone oil-based creams are effective in limiting the growth of scar tissue by increasing the humidity and local skin temperature of the scar epidermis (16–18). The application of silicone scar patches should be made as soon as the surgical wound has healed (2 weeks postoperatively). Cut the patch of scar slightly larger than the scar, and when the wound surface is fully epithelialized, a silicone gel may be used instead (19).

Compression therapy may prevent keloid elevation but is not as effective in keloid treatment compared with proliferative keloid therapy. One exception to this is the application of magnetic pressure ear clips following the excision of the keloid from the earlobe, which may inhibit keloid proliferation to some extent (2).

More recently, there has also been a growing amount of basic research devoted to the prevention of scar formation by exploiting the special properties of certain materials to fabricate hypotonic sutures, hypotonic dressings, and so on. Liquid-crystal elastomers (LCEs) are a class of soft active materials that are receiving increasing attention due to their excellent conductive and optical properties. Low-temperature synthesis methods using optimized composition ratios allow the LCE metamaterials to provide a reasonably high actuation stress/strain at substantially lower actuation temperatures (46°C). By combining this biocompatible LCE metamaterial with a medical dressing, a breathable, shrinkable, hemostatic patch can be developed and animal study species have demonstrated the benefits of this hemostatic patch and suture compared to conventional strategies (e.g., medical dressings and sutures) to speed up skin regeneration while avoiding the keloids (20).

In conclusion, surgical treatment remains the cornerstone of the treatment of hyperplastic scars, and in order to achieve optimal therapeutic results, it usually requires preoperative consideration of appropriate surgical options, intraoperative rational incision design, fine suturing, and postoperative combination with adjunctive therapies such as silicone scar patches or silicone ointments and compression therapy. Recent studies have also shown promise in the development of new materials for the prevention of scar formation. If the scar never improves, or if it is in an intimal location and the patient has fertility needs (e.g., perineum), it may be treated with injections of scar softening (e.g., tretinoin, a steroid inhibitor for scar reduction) or laser treatments (e.g., Nd: YAG 1064, fractional laser, pulsed dye laser, IPL, Q switched laser) as described below.

## 3. Physiotherapy

Physical therapy is a method of treating disease by utilizing various physical energies such as heat, light, and radiation, and it has many applications in the treatment of keloids. Physical therapy for keloids includes cryotherapy, photoelectric therapy, radiation therapy and photodynamic therapy. Cryotherapy can be used alone or in combination with surgery and local injections to reduce recurrence rates and improve esthetics and symptoms. Photoelectric therapy, which includes non-exfoliative photoelectric therapy and exfoliative photoelectric therapy, prevents and treats keloids by selectively acting at different depths of the skin. Radiation therapy prevents and treats keloids by inhibiting angiogenesis and fibroblast production. Photodynamic therapy treats keloids through the use of laser-activated photosensitizing drugs and is favored for its low toxicity and high selectivity. These physical therapies can be used either alone or in combination with other treatments to improve efficacy and minimize adverse effects.

### 3.1. Cryotherapy therapy

Cryotherapy is an effective treatment for keloids with a low rate of recurrence and may be applied alone or in combination with surgery and Intralesional injections to decrease recurrence rates and improve esthetics and symptoms (21). The mechanism of action is primarily vascular quiescence and the direct physical effect of cell freezing, where the direct physical effect of freezing cells refers to intracellular freezing, leading to disruption of cellular membranes and cell death; vascular quiescence refers to the process of freezing and thawing of cells, which results in tissue hypoxia and malnutrition, leading to focal cell death. Current methods of cryotherapy fall into two categories, contact cryotherapy (22) and Intralesional cryosurgery (23). Contact cryotherapy is typically used for hyperplastic keloids and keloids that are too small for a cryo-needle to be inserted, but multiple treatments are needed to flatten the keloids and can even lead to depigment of the patient’s superficial skin. In contrast, intralesional cryosurgery is a relatively new and safe treatment that can be used for any type and form of hyperplastic scars and keloids, particularly bulky keloids, and typically requires only a single treatment to flatten the patient’s keloids (23). Compared to Intralesional injections of tretinoin, 5-fluorouracil, and classical contact cryotherapy, intralesional cryosurgery causes minimal damage to the superficial skin and minimal pigmentation changes (24, 25), and its adverse effects primarily include depigmentation, recurrence, and pain, but in clinical trials pain and recurrence were uncommon and depigmentation was only of a temporary nature (26).

Cryotherapy may also be used in conjunction with surgery and Intralesional injections to further decrease recurrence rates and improve esthetics and symptoms (27–29). For example, combining cryotherapy with Intralesional steroid injections results in superior outcomes compared to both alone and is more effective in reducing keloid size; Litrowski has shown that the combination of surgery and cryotherapy is a valuable treatment for keloids that is effective, convenient, well-tolerated, and does not have any significant side effects beyond local pain and hyperalgesia (30); Azzam added platelet rich plasma (PRP) injections to combine all three, using cryotherapy during surgery, and showed that triple therapy could achieve improved efficacy, a lower rate of recurrence, good cosmetic outcomes, and the absence of significant side effects (31); Stromps combined the method of cryotherapy with silicone gel sheets and found that it had the potential to significantly reduce the size of the keloid and improve its hardness, pain, and discomfort (32).

### 3.2. Photoelectric therapy

At present, photoelectric treatment has been widely used in the prevention and treatment of scar. According to the depth and principle of its action on the skin, it is generally divided into non-exfoliative photoelectric treatment and exfoliative photoelectric treatment clinically.

#### 3.2.1. Non-exfoliative photoelectric therapy

Pulsed dye laser (PDL) is one of the first lasers used in the treatment of keloids, with the major application wavelengths of 585 nm and 595 nm. By selecting the blood vessels acting on the epidermis and the superficial dermis, PDL can destroy hemoglobin, reduce tissue capillaries, and inhibit the proliferation of fibroblasts (33). The Alster team first used 585 nm PDL to treat scars in the area of the sternum, with significant improvements in the height, elasticity, flexibility, and erythema of the keloid size by comparing the treated and untreated groups (34). The Manuskiatti team then had an in-depth discussion on the precision of the PDL treatment and found that there was no significant difference in the treatment results obtained with different energies, and that multiple effects in a short period of time could enhance the treatment effect (35).

Neodymium: yttrium aluminum garnet laser (Nd: YAG laser) wavelength is 1,064 nm. Compared with PDL, Nd: YAG is more suitable to solve the vascularization problem of keloids, which can also damage hair follicles and reduce hair follicle inflammation (36). The Chi Xu team used 585 nm PDL and 1,064 nm Nd: YAG to treat keloids between 4 and 6 weeks, and found that the flow perfusion of keloids could be significantly reduced, and the Nd: YAG laser combined with PDL was better (37).

The intense pulse laser IPL (Intense pulsed light) emits incoherent broadband wavelength pulse light and targets pigmentation and vasculature (38). The effect alone is not good, more combined with drugs, can relieve the thickness, erythema and pigmentation of keloids (39).

#### 3.2.2. Exfoliative photoelectric treatment

CO_2_ laser damages blood vessels and gasfies scar tissue while inhibiting fibroblast proliferation and inducing collagen remodeling (40), However, the recurrence rate within 2 years of treatment alone is extremely high, so it is mostly clinical combination therapy, such as TAC (41).

Compared with CO_2_ laser and Nd: YAG laser (Er: YAG laser), Er: YAG laser is also relatively effective, but skin scab and erythema are less time and less painful (42).

Keloid tissue is hard, and therefore drug injection is difficult. Radio frequency plasma (plasma) can dissociate the nitrogen molecules in the air into high-energy plasma states, and act on the dermis through heat energy to promote the rearrangement of collagen tissue rearrangement in the scar (43). It has a remarkable effect on the treatment of auricular keloid (44). RF is often used in combination with steroid drug injections to make the drug more easily absorbed (45). Compared with conventional intralesional RF, the Taneja team innovatively proposed to use the holes under the surface of the customized venous intubation to deliver energy during the RF process. Compared with conventional spot radiation energy, the energy can be dissipated in all directions, thus reducing the damage to the epidermis (46).

Regardless of the photoelectric treatment modality, it is important to reduce the inflammatory response after photoelectric therapy and prevent pigment changes. Therefore, it is often combined with auxiliary drugs that anti-inflammation and inhibit melanin formation, such as asiabside ointment (47).

### 3.3. Radiation therapy

Radiotherapy (RT) has been used to treat keloids for more than 100 years, mainly in preventing and treating keloids by inhibiting angiogenesis and fibroblast proliferation (48). RT is more suitable as postoperative adjuvant therapy than treatment alone. In a meta-analysis, postoperative RT was combined, but allowed reducing the recurrence rate after the surgery alone from 45–100% to less than 20% (49). In terms of treatment effect, the control of RT treatment dose and irradiation time is particularly important. The total recommended dose for treating keloids ranges from 12 to 20Gy, with the maximum biologically effective dose is 30Gy, but the degree of damage to visceral organs remains to be determined (50, 51). In addition to treatment modality, anatomical location is a key factor affecting the recurrence rate, the highest in the chest and lowest in the ear (49).

Radio therapy for keloids was first introduced in 1906 but 107 years later an optimal protocol has not been established. X-rays, beta rays, or gamma rays have been used in most studies. Heavy particle radiotherapy has been used in the clinical management of refractory and radiation-insensitive tumors in recent years, but its use in keloids has been infrequently reported. Chen’s team (52) carried out the first end-to-end particle radiotherapy after keloid surgery, and 16 patients were administered a total dose of 8Gy throughout the study, with an average follow-up of 29.7 months and a cure rate of 95%, and no patients experienced any complications. Heavy particle lineage therapy has been shown to have a precise killing effect on tumor cells by destroying the DNA double-strand of tumor cells, which can significantly reduce damage to normal tissues and organs with high efficacy and safety as well as achieve the ideal dose distribution and biologic effect. Thus, heavy particle lineage therapy could be a potential clinical treatment modality for keloids.

### 3.4. Photodynamic therapy

Because of the characteristics of Photodynamic therapy (PDT) low toxicity and high selectivity, is widely popular in clinical treatment, mainly through the use of laser activated photosensitive drugs, make heme bioconversion into protoporphyrin IX, the endogenous molecular oxygen into cytotoxic reactive oxygen species, direct damage to monocytes and macrophages, inhibit the inflammatory response to treat keloids (53). Clinically more used photosensitive drug is Zhuthemtin ointment (54). Several clinical trials have demonstrated that PDT is an important alternative to patients after ineffective steroid hormone therapy, but further evidence is needed due to the small number of clinical studies (55) (Table 1).

**Table 1:** Classification of keloid physical methods.

Therapeutic method	Mechanism of action	Effect efficacy
**Cryotherapy therapy**	Vascular quiescence, and the direct physical effect of cell freezing	Inhibits recurrence, improves pain and itching (21)
**Photoelectric therapy** Pulsed dye laser, IPL Nd: YAG laser, CO2 laser, RF, Er: YAG	Inhibits angiogenesis Inhibits fibroblast proliferation Promotes collagen remodeling Damages blood vessels and gasifies the scar tissue	Reduces height, improves erythema, vascularity, flexibility, color, and itching (33, 36, 38, 40, 42, 43)
**Radiation therapy**	Inhibits angiogenesis Inhibits fibroblast proliferation	Reduces height, improves vascular distribution, flexibility, and color (48–50)
**PDT**	Inhibits inflammatory response Inhibits fibroblast proliferation Promotes collagen decomposition Promotes epithelial tissue repair	There are fewer complications (53–55)

Overall, physical therapy offers a promising approach to the prevention and treatment of scars. However, the use of these physical therapies is still subject to some limitations, such as the efficacy of physical therapies may be limited by factors such as the type, size and depth of scars, as well as the possible adverse effects such as pain, itching, and hyperpigmentation that may occur during the treatment process. Therefore, future studies should further refine these physical therapies and explore new treatment methods to improve the efficacy and reliability of scar treatment.

## 4. Drug treatment

### 4.1. Glucocorticoid drugs

Triamcinolone (TAC) is the most commonly used hormone drug, mainly treating keloids with obvious “inflammatory” characteristics, which can effectively reduce the thickness of keloids, and relieve itching and pain (56). TAC as a monotherapy recurrence rate is as high as 50%, and up to 63% of patients will have postinjection side effects, such as telangiectasia, subcutaneous fat atrophy, pigmentation, Cushing’s syndrome (53), Combination therapy reduces side effects, such as RT (45), CO_2_ laser (41), 5-FU (57). In order to avoid the pain caused by glucocorticoid injection, steroid dressing is becoming more and more popular, Jinrong Li team also use electrospinning polymer microfiber dressing to treat keloids, which embedded with dexamethasone and antibacterial anti-inflammatory tea polyphenols, after 3 months of treatment, the size of keloid and erythema have improved, but also further reduce the risk of inflammation and infection (58).

### 4.2. Antitumor drugs

Keloids have the characteristics of tumor, so antitumor drugs also have a certain effect in the treatment of keloids. 5-Fluouracil (5-Fluorouracil, 5-FU) has direct cytotoxic effects that inhibit fibroblast proliferation, G2/M cell cycle arrest and apoptosis (59). In Tamoxifen (TAM), a selective estrogen receptor modulator used to treat breast cancer, it has been found that the average number of fibroblasts in keloids was significantly reduced after TAM treatment (60). Mitomycin C has antitumor activity and thus inhibits scarring by inhibiting DNA, RNA synthesis in fibroblasts (61).

### 4.3. Botulinum toxin type A

Botulinum toxin type A (BTX-A) is a potent neurotoxin that can cause rhabdomyolplegia. It plays a therapeutic effect in fibroblasts by inhibiting the proliferation of fibroblasts and TGF- β 1 expression (62). However, the Gauglitz team obtained different results, with the intratumoral injection of BTX-A lasting once every 1 month, and found that BTX-A had no effect on the proliferation and metabolism of fibroblasts, and could not improve the scarring. Although the treatment effect of the action alone is general, the preoperative injection can play a role in preventing the recurrence, and the adverse effects of the post-injection are relatively mild, and it can be self-recovered in a short time (63).

### 4.4. Immunomodulators

Tacrolimus as an mTOR receptor inhibitor can reduce histamine release, and effectively relieve itching, along with less skin absorption and a high safety profile (64). 5% Imiquimod ointment enhances cellular immune activity and suppresses keloid production by inhibiting collagen and glycosaminoglycan production (65). However, the effect is short and it will relapse completely after 4 weeks (66). Interferon (IFN) is an immunomodulator of glycoproteins that produces antifibrotic effects by interfering with collagen synthesis and fibroblast proliferation (5). In a clinical randomized controlled experiment, some scholars found that IFN can reduce the height of keloid, but due to the small clinical sample size, it is necessary to further explore the appropriate drug dose and action time of IFN (67).

### 4.5. Antihypertensive drugs

Angiotensin-converting enzyme inhibitors play an important role in collagen biosynthesis and wound healing (68). The formation of keloids involves the activation of the RAS system. Therefore, hypertensive drugs that antagonize the RAS system are an emerging treatment for keloid scars. They mainly include angiotensin-converting enzyme inhibitors (ACEI) and angiotensin II receptor antagonists (ARB). They inhibit excessive collagen formation in keloids and hypertrophic scars by antagonizing the RAS system. Ardekan’s team used 5% captopril cream for 6 weeks in a burn patient and found that it reduced the height of the keloid and improved redness and itching (69). In contrast to these results, Johanneke did not find that captopril improved scar formation in his experiments and only resulted in delayed wound closure (70). Chen found that the combination of captopril 10–2 mol/L and 5-FU 1 mg/mL reduced fibroblast proliferation and collagen deposition in keloid cultures, which was superior to captopril or 5-FU as a single therapeutic agent (71). Mohammadi et al. found that 1% enalapril significantly improved pruritus (72). Verapamil is a calcium channel antagonist that induces collagen degradation by inhibiting the inflammatory response and reducing ECM and collagen production (73). Verapamil was first given by Lawrence’s team for 7–14 days postoperatively and 52% of these patients recovered (74). Compared to TAC, side effects such as skin atrophy, capillary dilation, and hyperpigmentation were significantly lower with verapamil than with tretinoin, but weaker in terms of both flexibility and angiogenesis, and it can be seen that tretinoin is better than verapamil, while verapamil is safer (75). Hedayatyanfard found that losartan potassium ointment (5%) could treat keloid and hyperplastic scars through antifibrotic effects, with significant improvements in keloid scars on four indicators of vascular status, pigmentation, flexibility, and scar height (76). Zhao’s study found that a complex losartan cream (containing chitosan, cumene, and losartan) was effective in inhibiting scar formation by inhibiting the TGF-β/Smad pathway (77).

### 4.6. Other new organics therapy

#### 4.6.1. Vitamin A

The potential role of vitamin A and its derived forms in the treatment of keloids has been explored in numerous studies over the past several years. Retinoic acid and 9-cis-retinoic acid are two of these compounds that have received a great deal of attention. Retinoic acid is capable of ameliorating chronic inflammation in keloids by reversing the action of matrix metalloproteinases (MMP) and by preventing the expansion of keloid tissue in normal annular skin (78). Studies have also shown that silicone and retinoic acid creams can prevent and ameliorate hypertrophic scarring (79). Retinoic acid 9-cis is another compound that has received a great deal of attention as a potential treatment for pathological scarring. 9-cis retinoic acid has been found to increase HOXA5 expression, promote activation of the p53 signaling pathway, and inhibit proliferation, migration, and collagen synthesis in fibroblasts from pathological scar tissue while increasing apoptosis. 9-cis-retinoic acid may thus be a potential treatment strategy for pathologic scarring (80).

#### 4.6.2. Vitamin D

Vitamin D is a lipid-soluble vitamin that is widely found in nature and has been shown to play a significant role in human health. Keloid is a common skin disorder that is challenging to treat. A growing body of research in recent years has demonstrated that vitamin D is closely associated with keloid development and progression. For example, vitamin D deficiency could contribute to keloid development, and injection therapy with vitamin D can significantly decrease keloid thickness (81); vitamin D plays an important role in the inhibition of inflammation and fibrosis, which are two inevitable steps in keloid development and progression (82, 83); vitamin D plays an important role in the inhibition of inflammation and fibrosis, which are two inevitable steps in keloid development and progression. Some studies have also found lower levels of the vitamin D receptor (VDR) in keloids compared to normal skin, suggesting that VDR may play a role in keloid pathology (84). Several other studies have found that low serum concentrations of 25-hydroxyvitamin D and Koebnerisin can be used as potential markers to predict keloid tendencies (85).

#### 4.6.3. Natural plant organics therapy

Available studies have shown that some of the numerous natural compounds have the potential to inhibit the development of keloids, and unlike synthetic compounds, these natural compounds can avoid potential adverse effects when treating keloids, although, at the same time, they may act by affecting multiple signaling pathways. For example, quercetin is capable of attenuating keloid drug resistance to radiotherapy, with the primary mechanism of action being via inhibition of the phosphatidylinositol-3-kinase (PI3K)/Akt pathway negatively regulating hypoxia inducible factor 1 (HIF-1) (86); asiaticoside is capable of modulating extracellular matrix protein production and hindering invasive keloid fibroblast growth, in the former by downregulating TGF-β receptor expression at the mRNA level to upregulate Smad7 expression in a dose dependent manner (87), and the latter through the approach of inhibition of the GDF-9/MAPK/Smad signaling pathway (88); salvia extract inhibits fibroblast proliferation and invasion and reduces collagen synthesis, which is achieved through up-regulation of Smad7 expression, down-regulation of Smad2/3 phosphorylation level, and down-regulation of surviving protein in keloid processing (89, 90); and methoxycinnamine inhibits the deposition of collagen by limiting the induction of fibroblast collagen synthesis by TGF-β (91) (Table 2).

**Table 2:** Classification of keloid drugs and methods.

Therapeutic method	Mechanism of action	Effect efficacy
**Glucocorticoid drugs** Triamcinolone acetonide	Reduces fibroblast proliferation Reduces collagen production Inhibits the inflammatory response	Lower height, improves flexibility, redness, and relieves itching and pain (56)
**Antitumor drug** 5-Fluouracil, tamoxifen, mitomycin C	Reduces fibroblast proliferation Reduces collagen production Enhances apoptosis	Reduces the height, improves its flexibility, relieves itching, pain and other diseases (59–61)
**Botox type A**	Inhibits the fibroblast proliferation Enhances collagen decomposition	Improves pain and itching and induces apoptosis in fibroblasts (62, 63)
**Immunomodulator** Tacrolimus, 5% imiquimod cream, interferon	Inhibits angiogenesis Inhibits the inflammatory response Inhibits fibroblast proliferation Promotes collagen decomposition Reduces collagen production	Reduces the height, relieves itching, inhibits recurrence, focuses on prevention (64, 65)
**Dyazide** Angiotensin-converting-enzyme inhibitor, verapamil	Inhibits the fibroblast proliferation Reduces collagen production Promotes collagen decomposition Inducts collagen remodeling	Reduces height, improves vascular condition, pigmentation, and flexibility (68–70, 72, 73)
**Other new organics therapy** Vitamin A, vitamin D, natural plant organics therapy	Reducts fibroblast proliferation Enhances HOXA5 Reduces collagen production Enhances apoptosis	Improves chronic inflammation, prevents expansion into circumscribed normal skin, and reduces keloid thickness (78, 81)

In summary, a variety of drug therapies have been developed for the prevention and treatment of this disease; however, these therapies are associated with varying degrees of side effects. Combination therapies and novel organic therapies are expected to improve therapeutic efficacy while reducing adverse effects. Future research should focus on exploring the mechanism of action and therapeutic efficacy of novel organic therapies and developing effective combination therapies to improve the treatment of scar hypertrophy.

## 5. Biological therapy

### 5.1. RNA-based therapy

Current studies have shown that dysregulated noncoding RNAs (ncRNAs) have a significant role in the formation of keloids. Of these, three main types of ncRNAs, i.e., miRNA, lncRNA, and circRNA, have been reported to control fibroblast proliferation, migration, invasion, apoptosis, and collagen synthesis through different pathways (92), thereby participating in the keloidogenesis and developmental process. Some researchers consider miR-29a-3p to be a core miRNA that may play an important role in keloid development and progression and may therefore be an effective target for keloid therapy (93). Key processes involved in keloid regulation by lncRNA include the proliferation of fibroblasts, deposition of ECM (94), Wnt signaling (95), Hh signaling (96) and TGF-β signaling (97). CircRNAs in post-transcriptional biological functions, the differentially expressed circRNAs are primarily involved in apoptosis, adhesion to adherents patches, PI3K-Akt, and metabolic pathways (98). These include lncRNAs, circRNAs, which contain multiple miRNA binding sites, and adsorb miRNAs such as sponges, which act as competing endogenous RNAs (ceRNAs) and competitively bind miRNAs to regulate gene expression (99, 100).

We anticipate that ncRNAs will be potential diagnostic and therapeutic targets in the treatment of keloids, and additional studies are required to identify more effective strategies for keloid prevention and clinical treatment. Even though ncRNA-based therapeutic approaches offer some advantages over traditional small-molecule drugs, such as easier access to the target, the method of ncRNA drug delivery, and the issue of immune-related toxicity and other adverse effects must still be considered and addressed.

### 5.2. Cytokine-based therapy

#### 5.2.1. TGF-β

TGF-β is a naturally occurring multifunctional peptide that regulates gene expression through activation of the SMAD signaling pathway, thus affecting all stages of keloid wound healing. TGF-β receptor and ligand expression levels are significantly higher in keloid fibroblasts (KFs) compared to normal conditions and therefore TGF-β1 has been suggested to be one of the key players in the formation of keloids. At present, several drugs and compounds have been found to inhibit the TGF-β1 signaling pathway as well as to treat keloids. One of these, tacrolimus (FK506), a drug that inhibits TGF-β1-induced proliferation, migration, and collagen synthesis in keloid KFs, can effectively block the TGF-β/Smad signaling pathway through the downregulation of the TGF-β receptor (101). Furthermore, a lipocalin (ADP355) -based peptide is also capable of inhibiting TGF-β1-induced fibrosis in keloids and can potentially treat keloids through modulation of signaling pathways such as AMPK, SMAD3, and ERK. In xenograft mice, ADP355 has also been shown to significantly reduce total mouse keloid tissue weight, as well as the expression of pre collagen (102). Hederagonic inhibited the proliferation of KFs at a concentration of 50 nM, reduced the expression of TGF-β1-induced α-SMA and the production of type I procollagen, and decreased the migration of KF cells (103); compounds such as oleanolic acid (104), *glycyrrhiza glabra* (105), and loureirin A (106) were also found to regulate the proliferation and extracellular matrix deposition of keloid KFs by mediating the TGF-β1/Smad1 signaling pathway, with therapeutic potential for keloids. Based on the results of these drugs and compounds, inhibition of the TGF-β1 signaling pathway appears to be an effective approach to the treatment of keloids. However, further studies are still required to determine the safety and efficacy of these drugs and compounds to translate them into practical applications for the treatment of these diseases.

#### 5.2.2. Epidermal growth factor

Epidermal growth factor (EGF) has been shown to affect skin homeostasis and wound healing by regulating a variety of cellular functions of dermal fibroblasts. Fibroblasts isolated from keloids have been shown to produce 2 to 3 times the amount of collagen as fibroblasts from normal skin (107), so EGF could be one of the potential treatment options for keloids. Hyunbum found that exogenous EGF was able to increase the level of matrix metalloproteinase MMP-1, which induces collagen breakdown, and decrease lysyl oxidase (LOX) and 4 lysyl oxidase-like (LOXL), which synthesize collagen, thereby altering the remodeling process of ECM, which in turn improved the fibrotic phenotype of keloid dermal fibroblasts, as evidenced by the process of significant FSP-1, α-SMA, vimentin gene expression and vimentin protein expression decreased. Based on this, LOX and LOX-like family members could be seen as potential therapeutic targets for skin fibrosis and keloid tissue formation (108). In addition, Le found that silencing metalloproteinase protein 17 (ADAM17) may limit ECM deposition in keloid fibroblasts by inhibiting the activity of the EGFR/ERK pathway, thereby reducing proliferation, invasion, and migration (109).

### 5.3. Enzyme inhibitor

#### 5.3.1. Tyrosinase inhibitors

Tyrosinase inhibitors can inhibit the formation of keloids by targeting the Akt/PI3K/mTOR pathway, the MAPK/ ERK pathway. Thus, sunitinib may effectively inhibit keloid development through inhibition of the Akt/PI3K/mTOR pathway. Doses of 6 μM of this drug have been shown to significantly inhibit keloid fibroblast (KFs) proliferation, and doses in the range of 2.0 to 6.0 μM induce apoptosis in over 60% of keloid fibroblasts with no cytotoxicity to normal cell lines. In addition, sunitinib inhibited the migration and invasion of KFs and significantly reduced the levels of KFs collagen I and III at the mRNA and protein levels (110). Wang found that sorafenib was able to exert targeted inhibitory effects on the TGF-β/SMAD and MAPK/ERK signaling pathways, and may not only inhibit ECM proliferation, invasion, and production of KFs *in vitro*, but also exert inhibitory effects on KFs migration, angiogenesis, and collagen accumulation in keloid explants grown *in vitro* (111). Nintedanib is a receptor tyrosine kinase inhibitor targeting VEGF, PDGF, FGF, and TGF-β receptors, and it has been shown that when nintedanib is administered at doses between 1 and 4 μM, it inhibits cell proliferation, induces GO/G1 phase block, inhibits migration and invasion of keloid fibroblasts, and significantly inhibits the expression of type I and type III collagen in keloid fibroblasts (112). This study demonstrates the feasibility of tyrosinase inhibitors as one of the therapeutic options for the treatment of keloids.

#### 5.3.2. Heat shock protein

Heat shock protein (HSP) has been shown to promote wound healing by acting as a molecular chaperone and regulating the combined inflammatory and stress response during the wound healing process. Excessive levels of HSP, on the other hand, can enhance the inflammatory response and lead to an uncontrolled synthesis process. HSP plays a key role in keloid tissue. Studies have shown that the levels of hsp27, hsp47, hsp60, hsp70, and hsp90 are increased in keloid tissue compared to normal tissue (113). 17-AAG, an hsp90 inhibitor, inhibits the expression of Akt in fibroblasts, thereby suppressing their proliferation and reducing their migratory capacity, in addition to downregulating type I collagen mRNA and protein expression and inhibiting the TGF-β1/SMAD pathway, which has potential value for keloid therapy (114, 115).

### 5.4. Composition fat transplantation

Adipose-derived Mesenchymal Stem Cells (ADSCs) are cells with multi-directional differentiation potential and stem cell immune phenotype isolated from adipose tissue. They have become the research focus of keloid treatment because they can inhibit fibroblast proliferation and collagen synthesis, with convenient material source and little trauma (116). The extraction and culture process of ADSC is relatively complex, and the relevant research is still in the basic research stage and needs further clinical verification. Nano-fat grafting is a purely physical method of destroying mature adipocytes in adipose tissue and obtaining a celiac-like adipose tissue that preserves ADSCs and contains stromal vascular component cells, ECM, oil droplets, and swelling fluid. In recent years, many studies have reported that the internal scar injection of nano-fat can improve the scar hyperpigmentation, thickness, softness, and reduce the pain sensation (117). It has also shown that the therapeutic effect is not ideal, probably because of the relatively small ADSC content in the mixture during extraction (118). The stromal vascular component of fat is rich in stem cells and various growth factors, which has special regenerative potential, which mainly can treat keloids through growth factors that can promote wound healing, reduce inflammatory response and induce collagen remodeling (119, 120).

### 5.5. Platelet-rich plasma

Platelet-rich plasma (PRP) contains growth factors that can be involved in the different stages of wound healing (121). Some scholars did not respond to 17 patients with keloid resection after 4 TAC or RT injections, so PRP was injected every other month. After 3 injections, vascular hyperplasia, inflammation, pigmentation and flexibility were greatly improved, especially the pruritus condition improved significantly (122). Overall, PRP injection is a safe and effective adjuvant therapy (Table 3).

**Table 3:** Classification of keloid biological methods.

Therapeutic method	Mechanism of action	Effect efficacy
**RNA-based therapy** MiRNA, lncRNA, circRNA	Inhibits fibroblast proliferation and migration, and enhances apoptosis Inhibits collagen production	Inhibits keloid formation (92)
**Cytokine-based therapy** TGF-β, epidermal growth factor	Regulates gene expression Inhibits fibroblast proliferation Reduces extracellular matrix deposition Regulates skin fibroblasts Inhibits collagen production Promotes collagen breakdown	Enhances skin homeostasis and promotes wound healing (107)
**Enzyme inhibitor** Tyrosinase inhibitors, heat shock protein	Inhibits fibroblast proliferation Enhances apoptosis Inhibits angiogenesis Inhibits collagen accumulation	Inhibits keloid formation, improves inflammation, and ameliorates stress syndrome (112, 113)
**Component fat transplant** Fat stem cells Nano fat The stromal vascular component of the fat	Inhibits fibroblasts proliferation Reduces the inflammatory response Inducts collagen remodeling	Reduces height, improves color, flexibility, and relieves pain (116–120)
**Platelet-rich plasma**	Reduces the inflammatory response Reduces collagen synthesis Promotes collagen remodeling	Improves pigment, flexibility, and relieves itching (122)

In conclusion, keloids are a common and difficult to treat skin disease. Biologic therapies have made some progress as an emerging therapeutic approach. RNA-based therapies, cytokine therapies, enzyme inhibitors, constitutive fat grafts, and PRP have all been shown to be potentially valuable in the treatment of keloids. However, the safety and efficacy of these approaches need to be further investigated, especially in clinical applications. We hope that these biologic therapies will be studied and applied more intensively in future studies to provide more effective methods and strategies for the treatment of keloids.

## 6. Combined treatment

The recurrence rate of keloids is extremely high with monotherapy, and many local complications can occur. Therefore, the combination of multiple treatments is needed to reduce the recurrence rate, while improving the adverse effects caused by monotherapy approaches. According to the type of combination, it can be divided into double line therapy and triple line therapy.

### 6.1. Binary therapy

Intralesional injection of TAC is the most widely used treatment after kelkelectomy. There are clinical studies that show that after TAC + 5-FU for 12 months, the height, texture and congestion of keloid can be significantly improved (123). Meanwhile, TAC + verapamil was also shown to be effective and reduced the occurrence of telangiectasia and skin atrophy, which was statistically significant for the overall improvement of keloids (124, 125). In a reticular meta-analysis, The Sha Yang team compared cure rates across different treatment modalities, The results found that the highest treatment rate of TAC + BTX-A, TAC + BTX-A (82.2%) > TAC + 5-FU (69.8%) > BTX-A (67.3%) > 5-FU + A silicone (59.4%) > TAC + A silicone (58.3%) > 5-FU (49.8%) “bleamycin (42.0%) > TAC (26.7%)” Verapamil (26.2%) “Silicone (18.3%) (126). Meanwhile, the Hend D Gamil team also proved that the combination treatment of TAC and BTX-A was more effective than that of T AC and BTX-A, compared with the treatment alone (127). This shows that TAC is more effective than verapamil in monotherapy, and better tolerated than 5-FU and bleomycin, and results in maximizing cure rates when combined with other drugs.

In addition to combining drugs, there are many combinations of bitherapies. Drug and Laser Combination: Postoperative TAC + radiation/laser therapy (PDL, Nd: YAG, CO_2_) Ablation can prevent the hyperpigmentation due to the inflammatory response after the laser treatment (128–130). Combination of drugs and pressure treatment: In the treatment of auricular keloid, the Carvalhaes team injected TAC into the lesion once every month before surgery, and applied pressure earrings to the ear scar after surgical resection. The patient was well tolerated, and the recurrence rate was significantly reduced (131). Laser combined with laser: Ouyang explores PDL + CO_2_. The effect of laser treatment showed that the combination treatment was better than PDL treatment alone (132).

### 6.2. Triple therapy

Studies have been tried with two drugs + laser TAC + 5-FU + PDL (133) Or one drug plus two laser CO_2_ + PDL + TAC and other triple therapy to treat keloids (134). 5-FU + TAC + sodium hyaluronate was combined (135), Sodium hyaluronate can significantly reduce hyperpigmentation. Zeng et al. proposed “sandwich therapy,” namely preoperative radiotherapy, perforator flap transplantation of superficial iliac artery, postoperative radiotherapy for keloids, all flap survives well and has no serious complications (136). Triple therapy provides a multifaceted approach to the treatment of keloid, but it is difficult to determine the individual contribution of each treatment modality to the final outcome, and further clinical trials are validation (Table 4).

In general, the combination therapy idea mainly based on drug therapy has been widely adopted in clinical practice in recent years. By exploring the action characteristics of each therapy method and making up for the possible side effects of each therapy, it is possible so as to build a relatively complete treatment combination.

**Table 4:** Summary of keloid combination therapy.

Treatment category	Compound mode	Effect efficacy
Two combination therapy	TAC+5-FU	Improves the height, texture, and congestion condition (123)
	TAC+verapamil	Reduces the occurrence of telangiectasiasis and skin atrophy (125)
	TAC+BTX-A	Improves thickness and surface area (127)
Triple therapy	TAC+5-FU+PDL	Improves pigment, flexibility, and relieve itching (133)
	TAC+CO2+PDL	Relieves itch and pain (134)
	TAC+5-FU+sodium hyaluronate	Reduces pigmentation (135)
	Sandwich therapy	All flaps survived well and without serious complications (136)

## 7. Discussion

At present, for diagnosed keloids, intralesional corticosteroid injections are used as a first-line treatment either as monotherapy or in combination with other modalities. Primarily devoted to adjuvant therapy following surgical excision, TAC is also a good conservative treatment when the patient is unsuitable for postoperative excisional radiation therapy, e. g. in the perineum. It is recommended that the optimal interval between injections be 2 weeks. In a systematic review published in Laura A. Walsh, other methods of intralesional injection are compared, including botulinum toxin A (BTA), bleomycin, mitomycin C, PRP, and collagenase. Verapamil may be treated both as an intra-focal injection and as an adjunctive treatment to cryotherapy or resection, and while its therapeutic efficacy may not always be superior to TAC, the drug is well tolerated and the potential for adverse effects may be lower. It has been shown that 5-FU can be used in conjunction with TAC therapy to prolong synergy but also increases the risk of ulcer development. Bleomycin is not as effective as TAC and has an increased risk of macrosomia and ulceration. BTA is superior to 5-FU alone and is no different from TAC in a double-blind study with a lower risk of hypopigmentation. BTA is a potent antihypertensive agent. Surgical excision combined with adjuvant cryotherapy and PRP resulted in a recurrence rate of 16.21%, but as there was no control group in this study, further clinical studies are required to further confirm the therapeutic role of PRP. Phototherapy (most commonly PDL or ablative laser therapy) has been advocated as a second-line treatment after surgical excision, and laser-assisted corticosteroid administration and the combined use of different lasers for keloid scarring are emerging treatments.

Treatment of keloids ranges from pre-surgical prophylaxis to post-surgical treatment and from pharmacologic therapy to physiotherapy, which are all currently clinically covered, but none appear to guarantee treatment response and prevent relapse. This is mainly due to the lack of a consistent control group across the many studies, which makes it difficult to compare results across studies. Heterogeneity in subject characteristics such as family history, keloid location, skin tone, size, and number as well as sex and skin type may also play a role in keloid formation. Recent research into new therapies has shown some promising results such as heavy particle therapy, antihypertensives, vitamins, biologic agents, etc. Again, however, the number of studies that have been confirmed in clinical research is relatively low, and most of them are in the basic experimental phase, so comparisons are difficult. There is a need to systematize the treatment of keloid scars, with the accurate diagnosis of keloid scars before treatment, all-around prevention before, during, and after surgery, management of patients’ habits, and exploration of the mechanism of keloid scar formation.

## 8. Summary

In recent years, new treatment methods of keloid have emerged in an endless stream, and surgical therapy, physiotherapy and hormone, anti-tumor and other drug therapies have been widely used in clinical practice. Other new drug therapies cover a wide range of mechanistic pathways and have good clinical research prospects. However, many experiments have been limited to animal experiments or randomized controlled experiments with small sample sizes, making it necessary to conduct more diverse clinical studies need to further determine the optimal time and dose of treatment. Since the onset of keloid is also closely related to the patient’s individual constitution, personalized, comprehensive treatment is the key to achieving the optimal results.

## Author contributions

WQ: Writing – original draft, Writing – review & editing. XX: Writing – original draft, Writing – review & editing. JT: Writing – review & editing. NG: Writing – review & editing.
